# On the feasibility of ultrasound Doppler-based personalized hemodynamic modeling of the abdominal aorta

**DOI:** 10.1186/s12938-024-01267-3

**Published:** 2024-07-25

**Authors:** Judith Fonken, Milan Gillissen, Eline van Engelen, Marc van Sambeek, Frans van de Vosse, Richard Lopata

**Affiliations:** 1 Photoacoustics & Ultrasound Laboratory Eindhoven (PULS/e), Eindhoven University of Technology, Groene Loper 3, Eindhoven, 5612AE The Netherlands; 2 Department of Vascular Surgery, Catharina hospital, Michelangelolaan 2, Eindhoven, 5623EJ The Netherlands; 3 Cardiovascular Biomechanics, Eindhoven University of Technology, Groene Loper 3, Eindhoven, 5612AE The Netherlands

**Keywords:** Abdominal aortic aneurysms, Ultrasound Doppler, Hemodynamics, Personalized modeling, Computational fluid dynamics

## Abstract

**Background:**

Personalized modeling is a promising tool to improve abdominal aortic aneurysm (AAA) rupture risk assessment. Computed tomography (CT) and quantitative flow (Q-flow) magnetic resonance imaging (MRI) are widely regarded as the gold standard for acquiring patient-specific geometry and velocity profiles, respectively. However, their frequent utilization is hindered by various drawbacks. Ultrasound is used extensively in current clinical practice and offers a safe, rapid and cost-effective method to acquire patient-specific geometries and velocity profiles. This study aims to extract and validate patient-specific velocity profiles from Doppler ultrasound and to examine the impact of the velocity profiles on computed hemodynamics.

**Methods:**

Pulsed-wave Doppler (PWD) and color Doppler (CD) data were successfully obtained for six volunteers and seven patients and employed to extract the flow pulse and velocity profile over the cross-section, respectively. The US flow pulses and velocity profiles as well as generic Womersley profiles were compared to the MRI velocities and flows. Additionally, CFD simulations were performed to examine the combined impact of the velocity profile and flow pulse.

**Results:**

Large discrepancies were found between the US and MRI velocity profiles over the cross-sections, with differences for US in the same range as for the Womersley profile. Differences in flow pulses revealed that US generally performs best in terms of maximum flow, forward flow and ratios between forward and backward flow, whereas it often overestimates the backward flow. Both spatial patterns and magnitude of the computed hemodynamics were considerably affected by the prescribed velocity boundary conditions. Larger errors and smaller differences between the US and generic CFD cases were observed for patients compared to volunteers.

**Conclusion:**

These results show that it is feasible to acquire the patient-specific flow pulse from PWD data, provided that the PWD acquisition could be performed proximal to the aneurysm region, and resulted in a triphasic flow pattern. However, obtaining the patient-specific velocity profile over the cross-section using CD data is not reliable. For the volunteers, utilizing the US flow profile instead of the generic flow profile generally resulted in improved performance, whereas this was the case in more than half of the cases for the patients.

## Background

A localized dilation of the infrarenal aorta, an abdominal aortic aneurysm (AAA), often presents without symptoms. However, it can progressively enlarge until it ruptures, resulting in an alarming 80% mortality rate [1, 2]. Surgical intervention is a viable option to prevent rupture, but it carries its own set of risks [2]. Consequently, assessing and monitoring the patient’s risk of rupture over time is crucial. Current clinical guidelines solely rely on the maximum diameter and growth rate of the aneurysm for the estimation of the rupture risk, based on findings from randomized clinical trials [3, 4]. Nonetheless, it is suggested that examining wall mechanics and hemodynamics could provide more precise risk indicators [1, 2].

Several studies have utilized computational solid and fluid dynamics models to investigate various parameters, including peak wall stress and wall shear stress (WSS). Peak wall stress is considered a crucial parameter for predicting aneurysm rupture, while low and disrupted WSS is believed to contribute to the formation of intraluminal thrombus (ILT) [1, 5–13]. However, in vivo, the deformation of the AAA wall is influenced by the hemodynamics within the AAA, and conversely, the wall deformation affects the hemodynamics. To incorporate this mutual influence in AAA simulations, fluid–structure interaction (FSI) models are necessary.

A comprehensive longitudinal study is imperative for a better understanding and prediction of AAA development, growth, and rupture risk. Given the significant influence of both wall mechanics and hemodynamics on AAA geometry, a patient-specific assessment is indispensable [1, 6]. Computed tomography (CT) remains the gold standard for acquiring patient-specific AAA geometries. However, frequent CT scans are hindered by the use of contrast agents and radiation exposure [1]. Another possibility is magnetic resonance imaging (MRI), although it is burdened by prolonged scanning periods and substantial costs. Previous FSI studies have either relied on idealized AAA geometries [14–16] or a limited number of CT- or MRI-derived geometries [7, 17–19], precluding large, longitudinal analyses using FSI simulations. Time-resolved three-dimensional ultrasound (4D US) emerges as the preferred imaging modality for extracting patient-specific geometries. Notably, 4D US is safe, rapid, cost-effective, and offers both temporal geometric and functional information [20–22]. Furthermore, ultrasound is already integrated into current clinical workflows, enhancing its feasibility for longitudinal AAA studies.

Although 4D US data are limited in terms of contrast with respect to CT, recent improvements in segmentation methods allow for the use of 4D US data in CSS and FSI models [5, 20, 21, 23], showcasing good correspondence between 4D US-based and CT-based segmentations and resulting wall stresses [5, 21]. Previous research also showed that the limited field-of-view of 4D US can be extended using multiperspective US, in which proximal and distal US acquisitions are fused [24, 25]. Still, the aorto-iliac bifurcation is hard to detect with ultrasound, due to the depth and tortuosity of the iliac arteries [26]. It has been shown that the absence of the bifurcation does not significantly influence the numerical assessment of the AAA wall mechanics [26] and that a parametric bifurcation can be added to the aneurysm geometry without significantly affecting the AAA hemodynamics [27].

Additional functional information can be extracted using US, enabling further personalization of the AAA Finite Element (FE) models. Speckle-tracking methods can be employed to obtain the patient-specific shear modulus [20], whereas US Doppler imaging can be utilized to obtain the patient-specific velocity profiles, both over time and over the cross-section [28, 29]. Previous studies utilized generic velocity profiles [23, 27]. However, it has been demonstrated that substantial variations in velocity profiles exist among patients [29], and these profiles exert a significant impact on the computed hemodynamics [30–32]. Quantitative flow (Q-flow) MRI is considered the gold standard to obtain patient-specific velocity profiles, but its routine clinical use is hampered by its lengthy scan duration and high costs [33–35].

This study aims to extract and validate patient-specific velocity profiles from US Doppler data. To this end, pulsed-wave Doppler (PWD), color Doppler (CD) and Q-flow MRI data were obtained for healthy volunteers and AAA patients. US-based and generic velocity profiles were compared to the MRI-derived velocity profiles for validation and computational fluid dynamics (CFD) simulations were analyzed to assess the impact of the prescribed velocity profiles on the computed hemodynamics.

## Results

### Velocity profiles over cross-section

**Fig. 1:**
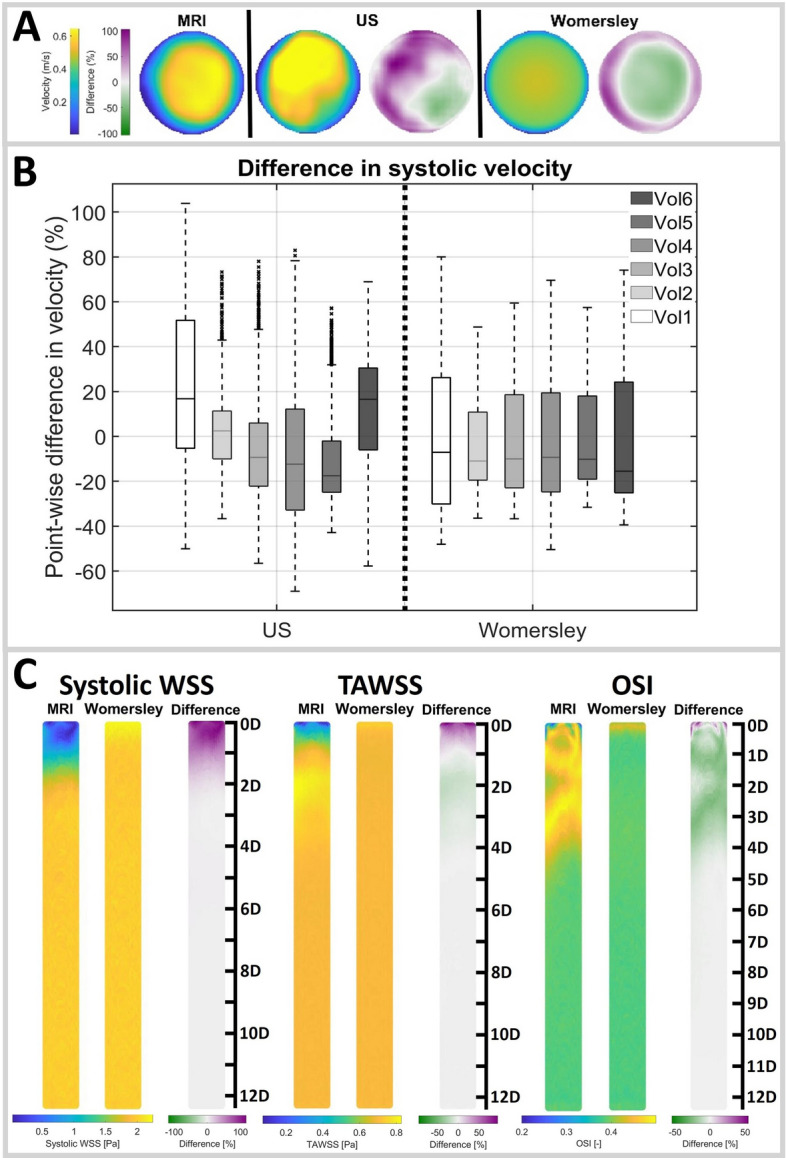
**A** Systolic MRI, US and Womersley velocity profiles over the cross-section (blue-yellow), and their percentual differences (green-purple), for a representative volunteer. **B** Percentual point-wise differences in velocity w.r.t. MRI for all volunteers for the US (left) and Womersley (right) velocity profiles. **C** Influence of the velocity profile on the systolic WSS, TAWSS, and OSI for a representative volunteer. The scale on the right indicates the length as multiple of the diameter (D)

Figures 1A and S2 show the MRI, US, and Womersley systolic velocity profile over the cross-section and their point-wise differences with respect to MRI. These results illustrate poor resemblance to MRI for the US profiles, since both the velocity magnitude and the spatial velocity pattern deviates. This deviation is observed for all volunteers, and it varies among individuals, which complicates attempts to correct for these discrepancies. For all volunteers, the Womersley profiles appear slightly flatter than the MRI profiles, overestimating the velocities at the borders while underestimating the velocities in the center of the vessel.

The point-wise differences in velocity with respect to MRI for all volunteers are summarized in Fig. 1B, illustrating similar median differences for US and Womersley. However, the range in differences is generally larger for US. Additionally, a larger variety in differences is observed between the volunteers for US.

To investigate the influence of the velocity profile on the computed hemodynamics, CFD simulations were performed and the result for a representative volunteer can be found in Fig. 1C. The differences in hemodynamics are largest at the inlet, but reduce considerably over the length of the vessel. The impact of the velocity profile on the OSI is most pronounced. After a length of 3 times the diameter, the nRMSE of both systolic WSS and TAWSS is below 10% for all volunteers, whereas a length of six times the diameter is needed to drop the nRMSE for the OSI below 10% (Fig. S3).

**Fig. 2:**
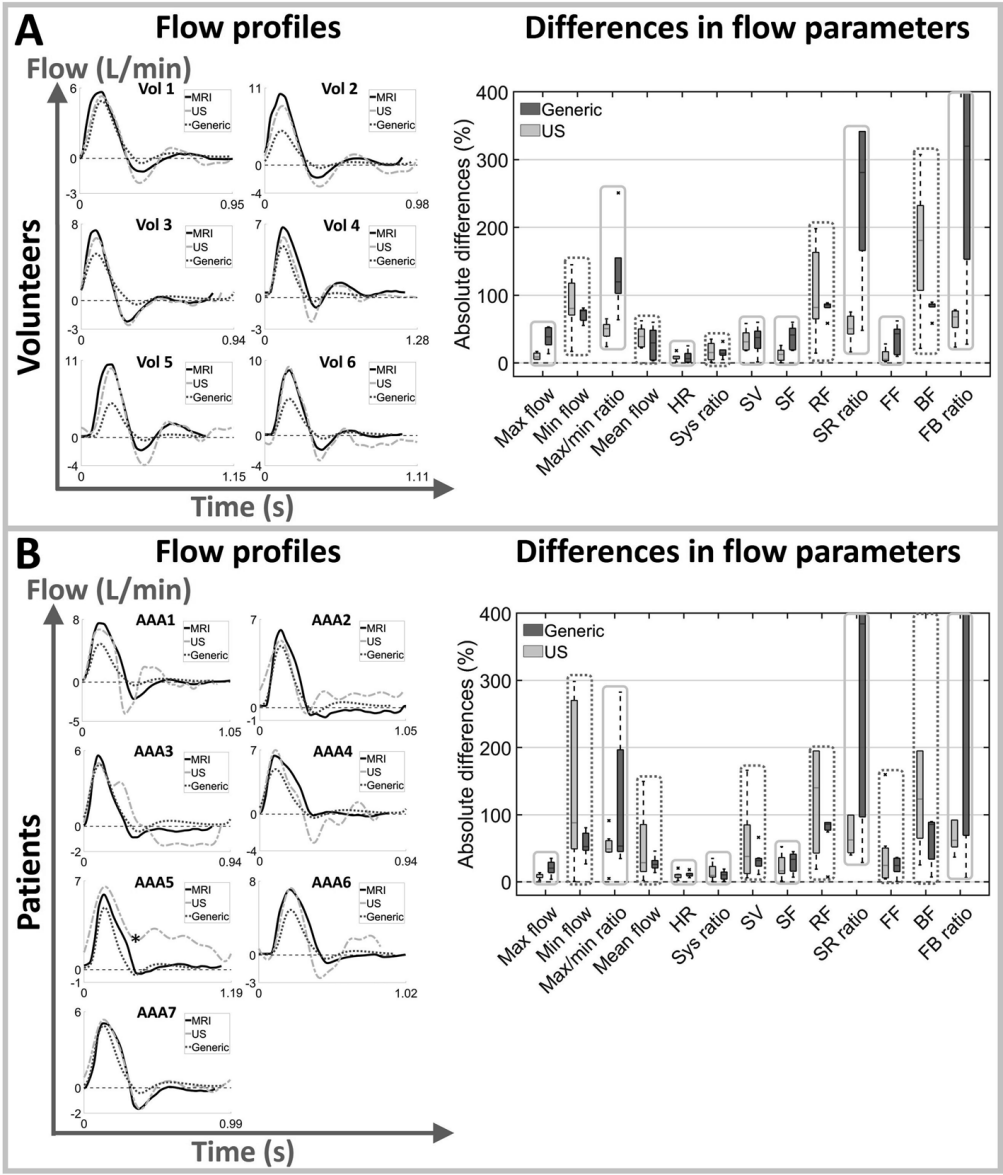
Flow profiles (left) and absolute percentual differences in flow parameters (right) for volunteers (**A**) and patients (**B**). The boxes in the boxplot (right) indicate the flow pulse that best approaches the MRI flow pulse, based on the median difference: US (solid) or generic (dashed). For visualization, the y-axis was trimmed to 400%

### Flow pulses

Various flow parameters were calculated to characterize the flow pulses (Fig. 7A). The left column of Fig. 2 shows the MRI, US, and generic flow profiles for all volunteers (A) and patients (B), respectively.

For all participants, the maximum flow as measured with MRI was best matched with US, although the difference between US and generic profiles was less pronounced for the patients. The maximum flow ranged between 5.7$-$10.4 L/min and 5.1$-$7.5 L/min for the volunteers and patients, respectively. For the generic profile, the maximum flow equalled 5 L/min, whereas for US, the maximum flow varied between 5.3$-$9.3 L/min (volunteers) and 5.0$-$7.2 L/min (patients).

For all volunteers, the flow pulse exhibits a triphasic pattern, which is typical for the abdominal aorta [12, 29, 36]. For two patients (AAA3 & AAA5), no triphasic flow was observed for the US profile. In one of these patients (AAA5), no negative flow was observed. For this patient, the systolic time (*E* in Fig. 7A) was manually selected (indicated with an asterisk in Fig. 2B) and all parameters including negative flow were left out of the comparison (indicated as NaN). Additionally, for two MRI-based flow pulses (AAA2 & AAA3), no second zero-crossing was observed, leading to similar values for various parameters (SF = FF, RF = BF, SRratio = FBratio).

The differences in flow parameters for all volunteers and patients are summarized in the right column of Fig. 2. The US flow profile generally has the lowest differences in the heart rate and in the parameters describing forward flow and ratios between forward and backward flow (maximum flow, max/min ratio, SF, SRratio, FF, and FBratio). The generic profile typically performs better for the mean flow and the parameters describing reversed flow (minimum flow, RF and BF). For the systolic ratio and the SV, no clear differences were observed between the US and generic flow pulse. Compared to the volunteers, the US flow pulse for patients resulted in larger differences for the minimum flow, mean flow, SV, SF, RF, and FF.

### Hemodynamics

**Fig. 3:**
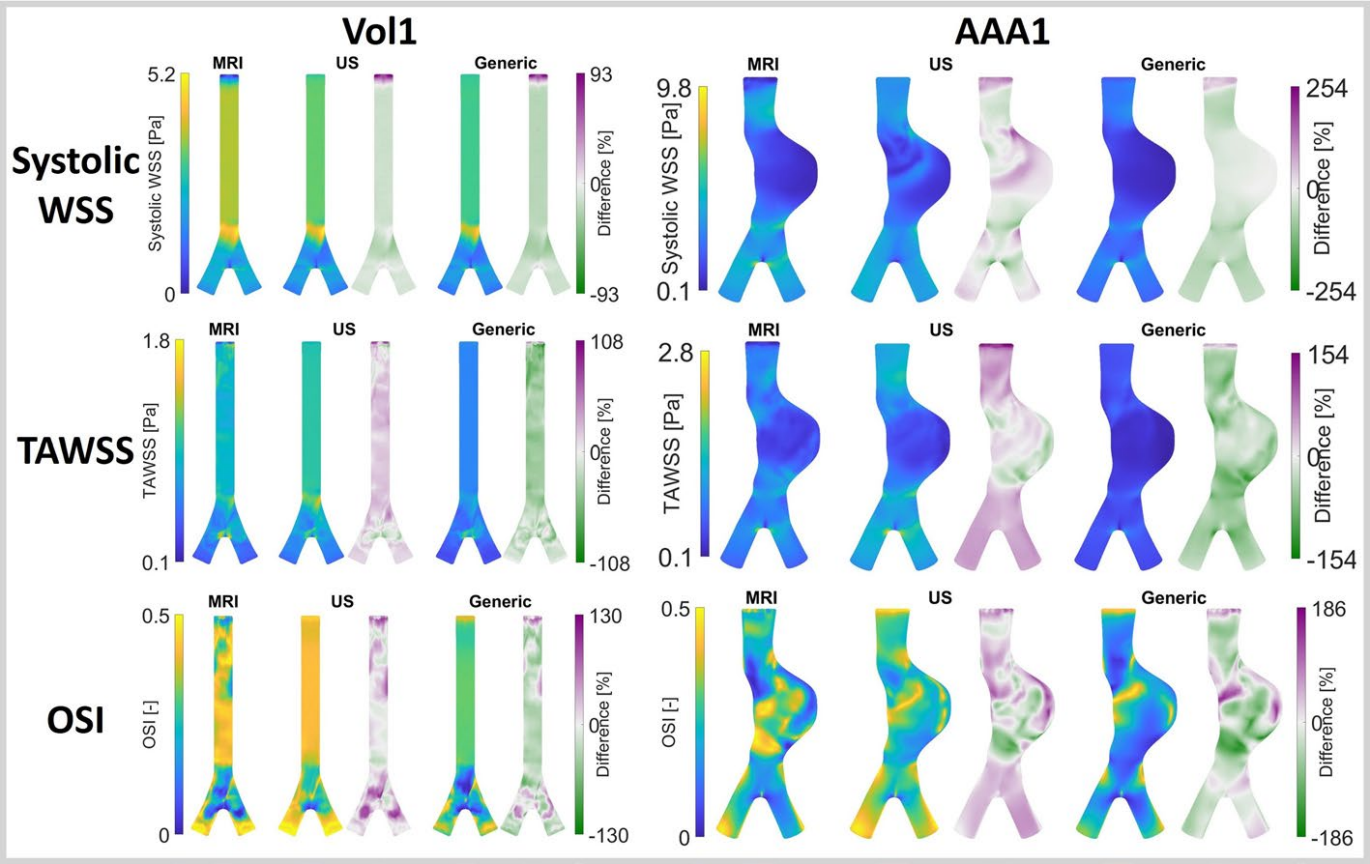
MRI, US and generic computed hemodynamics and their percentual differences, for a representative volunteer (left) and patient (right)

**Fig. 4:**
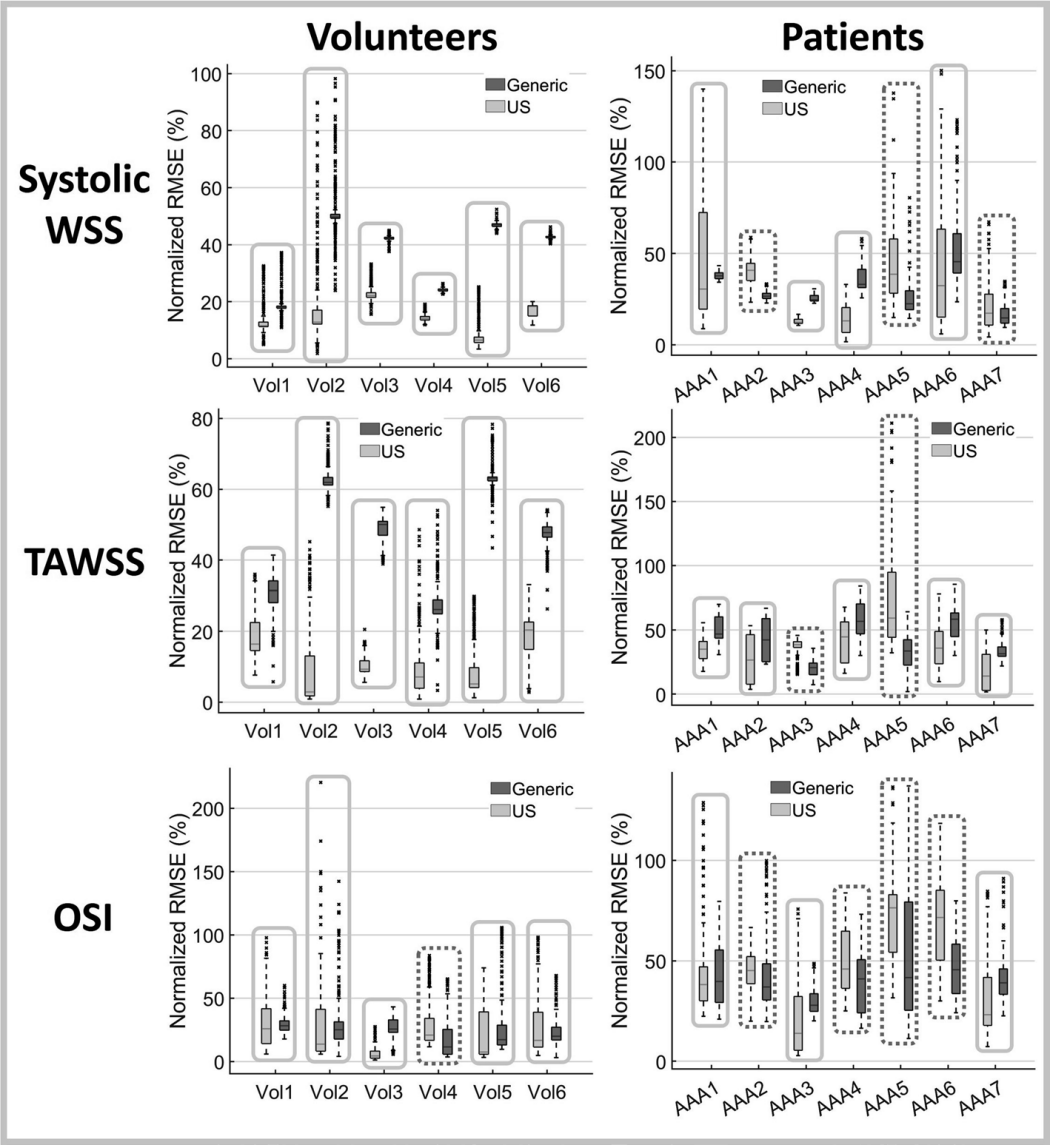
Boxplots displaying the nRMSE in hemodynamics for volunteers (left) and patients (right). The most proximal part of the domain, equal to one diameter, was excluded. The boxes indicate the simulation with the lowest median nRMSE: US (solid) or generic (dashed)

The combined effect of the flow pulse and velocity profile over the cross-section was evaluated by simulating three different CFD cases for each participant (Fig. 7B). The resulting systolic WSS, TAWSS, and OSI patterns for a representative volunteer and patient are shown in Fig. 3. For the volunteers, the largest differences in spatial pattern are found near the inlet, similar as observed in Fig. 1C. The maximum percentual point-wise differences range between 65–194% (systolic WSS), 77–220% (TAWSS), and 78–147% (OSI). More distal in the domain, the differences in spatial patterns, especially for the systolic WSS and TAWSS, diminish. Nevertheless, variations in magnitude persist. For the patients, the differences in spatial patterns are more pronounced and persist throughout the complete domain. Furthermore, larger differences in magnitude are observed, with maximum differences ranging between 133–435% (systolic WSS), 145–565% (TAWSS), and 122–247% (OSI).

Figure 4 summarizes the nRMSE for all hemodynamic quantities for both volunteers and patients. For the systolic WSS and TAWSS, the US case results in considerable lower nRMSE than the generic case for all volunteers. The median nRMSE ranged between 6.5$-$22.2% (systolic WSS) and 2.9$-$20.4% (TAWSS) for the US case, compared to 17.9$-$49.7% (systolic WSS) and 26.1$-$63.1% (TAWSS) for the generic case. For the patients, the differences between the US and generic case are less evident and the nRMSE is larger, with median values varying between 12.3$-$40.7% (US) and 22.4$-$45.4% (generic) for the systolic WSS and between 26.5$-$59.2% (US) and 20.6$-$58.4% (generic) for the TAWSS. Based on the median nRMSE, the US case performs better for 4 (systolic WSS) and 5 (TAWSS) out of 7 patients.

For the OSI, the US case yields the lowest RMSE for all volunteers except one, with median values fluctuating between 4.4$-$25.8% (US) and 11.5$-$28.2% (generic). Again, for the patients, the differences between US and generic are less apparent, with medians ranging between 13.9$-$76.4% (US) and 27.9$-$45.5% (generic). The US case results in slightly lower median RMSE for 3 out of 7 patients.

## Discussion

This study demonstrated that personalizing CFD simulations of AAAs is feasible using PWD. Flow pulses were successfully extracted from PWD data and compared to MRI and generic profiles (Sect. Flow pulses). US generally performs best for estimating parameters describing forward flow and ratios between forward and backward flow, whereas it often overestimates the backward flow. Furthermore, the superior performance of US was less pronounced for the patients. Multiple factors can account for this difference between patients and volunteers. Firstly, the maximum flows were typically lower among patients compared to volunteers, aligning more closely with the generic maximum flow. Secondly, the PWD imaging depth generally was larger for the patients than the volunteers (Table 1), leading to impaired image quality due to attenuation of the US signal. Additionally, in two patients, the PWD data could not be obtained proximal to the aneurysm region, due to spatial obstruction by the ribs. Flow pulses obtained in the aneurysm region may not be representative for the situation proximal to the aneurysm and can therefore not be used to prescribe the inlet flow in hemodynamic simulations. Lastly, the abdominal aortic flow pulse typically exhibits a typical triphasic pattern [12, 29, 36], which was also observed in the PWD measurements for all volunteers and most patients. However, for two patients, no triphasic flow pulse was observed. Furthermore, for two MRI datasets, no second zero-crossing was present. Both deviations in shape may cause inaccurate flow parameter values, especially for the reversed flow and ratios between forward and backward flow. In future studies, patients should be excluded if a proximal PWD measurement is impossible or if the extracted flow pulse lacks the typical triphasic pattern.

Obtaining the patient-specific velocity profile over the cross-section using CD data is not feasible, as the errors obtained with respect to MRI are comparable to those for the Womersley profile. The poor performance of CD can be explained by several limitations. Since CD data are obtained over a two-dimensional area, whereas PWD data are obtained over a single line, CD sensitivity and accuracy are impaired. Furthermore, the CD velocity measurements are highly angle dependent [37–39]. Lastly, CD measurements can be hampered by inferior US quality due to large depth, intestinal function and bowel gas.

The spatial patterns for the computed hemodynamics were considerably affected by the prescribed velocity profiles, especially at the inlet of the domain, consistent with findings in literature [31]. The greatest impact was observed for the OSI, which can be explained by the difference in skewness of the velocity profiles, creating differences in oscillatory behavior of the WSS. For the volunteers, the differences in spatial pattern vanish over the length of the vessel, especially for the systolic WSS and TAWSS, whereas they persist for the patients. This disparity can be attributed to the incorporation of the patient-specific aneurysm geometry for the patients, compared to a simple, straight vessel for the volunteers. The geometry has a large impact on the computed hemodynamics [23], mainly due to flow impingement on the vessel wall. However, to assess the differences in hemodynamics for realistic CFD simulations of AAAs, it is deemed necessary to utilize the patient-specific geometry instead of a generic one. Furthermore, differences in velocity profiles result in considerable differences in systolic WSS, TAWSS, and OSI magnitude. Median nRMSE ranged between 2.9$-$25.8% (US) and 11.5$-$63.1% (generic) for volunteers and between 12.3$-$76.4% (US) and 20.6$-$58.4% (generic) for patients, showcasing larger nRMSE and smaller differences between US and generic for patients. Utilizing the US flow profile instead of the generic one resulted in improved performance with respect to MRI for all volunteers and more than half of the patients.

Apart from differences between imaging modalities, differences in flow pulses may be caused by differences in blood pressure (Table 1), acquisition location, and acquisition timing. Prior to the US acquisitions, participants fasted for a minimum of 4 h to reduce intestinal function and bowel gas. No fasting was required prior to the MRI measurement, possibly leading to differences in blood distribution to different parts of the body. Furthermore, the mean velocity was extracted from PWD over a single diameter of the vessel. To calculate the flow, a symmetric velocity profile and a perfect circular cross-sectional area were assumed, which may cause inaccuracies in flow pulse.

Q-flow MRI imaging is widely acknowledged and used as ground-truth [30, 33, 40], but suffers from drawbacks as well. Several measurement errors, due to the lower spatial and temporal resolution, low velocity SNR, phase dispersion, partial volume effect and slice positioning, may lead to uncertainties of 5–10% in flow [34, 41, 42].

For both US Doppler as Q-flow MRI, only the through-plane components of the velocities could be obtained. However, previous research has shown that the in-plane velocities are small and have insignificant influence on the hemodynamics [30]. Additionally, the breath holding procedure during both US and MRI acquisitions may affect the flow conditions. Nevertheless, the same procedure was used for both modalities and previous studies have shown that breath-hold with small lung volume by shallow inspiration, as used in this study, has a negligible effect on the measured velocities [43, 44].

One of the major limitations of this study is the limited number of participants, due to the high costs and low availability associated with MRI scans. Future studies should focus on extending the number of participants, preferably in a multi-center study. Furthermore, future studies should focus on ultrasound vector flow imaging (VFI), which represents an emerging technology to overcome the limitations of conventional Doppler imaging [39].

## Conclusions

This study demonstrated that obtaining the patient-specific flow pulse is feasible using pulsed-wave Doppler ultrasound, provided that the PWD acquisition is performed proximal to the aneurysm region and results in a realistic, triphasic flow pattern.

Obtaining the patient-specific velocity profile over the cross-section using CD data is not reliable, as the errors obtained with respect to MRI are comparable to those for the Womersley profile.

Computed hemodynamics were considerably affected by the prescribed velocity profiles, both in magnitude and spatial patterns. Larger errors with respect to MRI and smaller differences in errors between the US and generic cases were observed for patients, compared to volunteers. Employing the US flow profile instead of the generic one resulted in improved performance for all volunteers and more than half of the patients.

## Methods

### Data acquisition

US and MRI datasets were acquired for six healthy volunteers and eight AAA patients in the Catharina Hospital in Eindhoven. All participants gave their written informed consent and the study was approved by the local ethics committee. One patient was excluded due to poor US image quality. The characteristics of the resulting participants are summarized in Table 1.

**Table 1:** Characteristics of all participants, including gender (male: M or female: F), age, number of days between US and MRI acquisitions, US and MRI diastolic (dia) and systolic (sys) blood pressure (BP), the PWD imaging depth and overview of employed scans: 1—CD, 2—PWD, 3—2D+t MRI, 4—4D US, 5—4D MRI. *DM* data missing

	Gender	Age	Days between US and MRI	BP US (mmHg) [dia sys]	BP MRI (mmHg) [dia sys]	Imaging depth PWD (cm)	Employed scans
Vol1	F	25	0	DM	[55 103]	3.5	1,2,3
Vol2	M	27	0	[70 132]	[58 114]	3.5	1,2,3
Vol3	M	56	0	[71 130]	[64 115]	8.5	1,2,3
Vol4	F	53	0	[74 107]	[55 119]	3	1,2,3
Vol5	M	27	0	[81 137]	[56 107]	3	1,2,3
Vol6	M	64	0	[77 133]	[61 121]	4	1,2,3
AAA1	M	77	0	[87 142]	[71 120]	6	2,3,4
AAA2	M	79	2	[84 167]	[73 158]	8	2,3,4
AAA3	M	80	0	[101 182]	[70 134]	6	2,3,4
AAA4	M	83	1	[90 170]	[94 149]	10	2,3,4
AAA5	M	72	4	[67 128]	[59 111]	7	2,3,4
AAA6	M	68	43	[76 116]	[64 114]	7	2,3,5
AAA7	F	68	3	[91 149]	[77 125]	9	2,3,4

#### Ultrasound

**Fig. 5:**
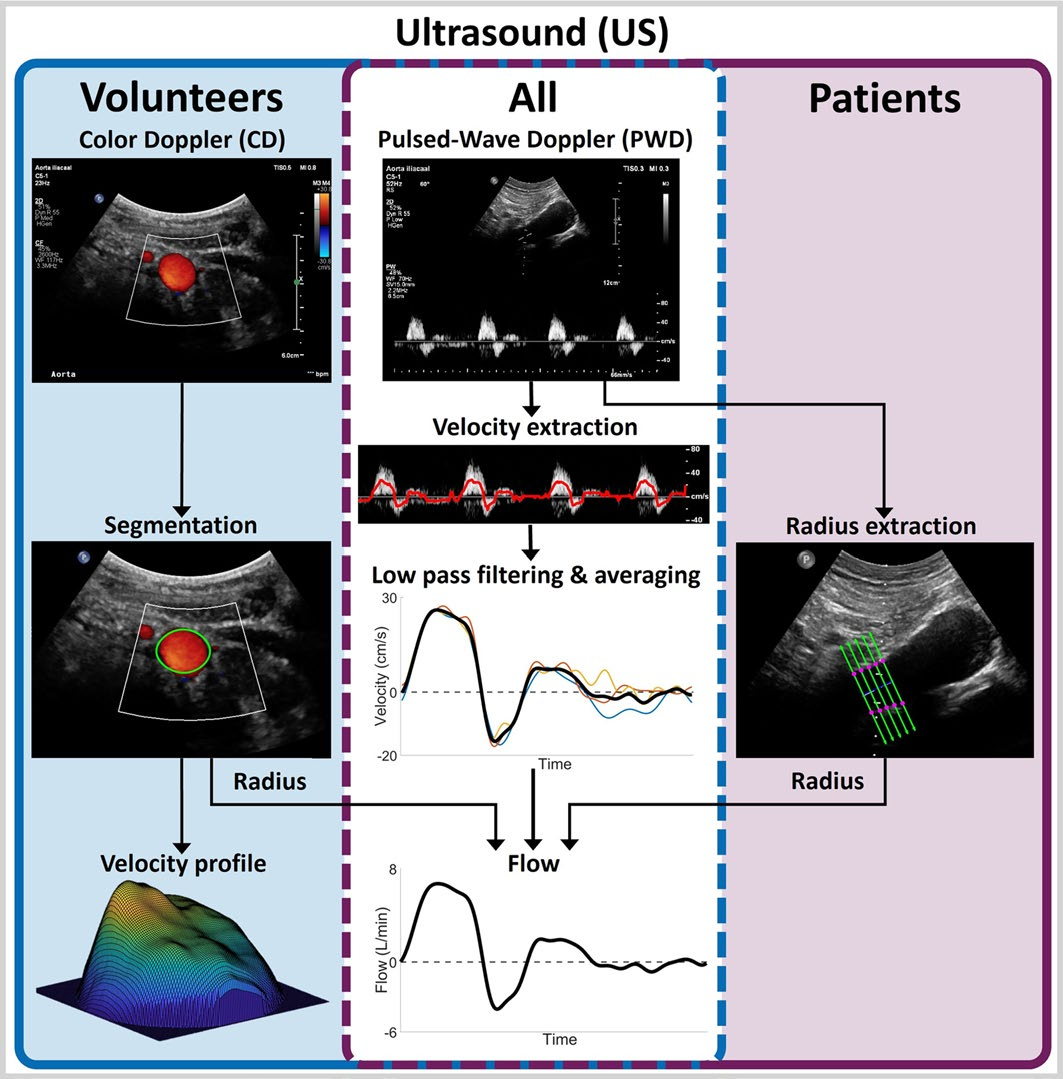
Illustration of the method to extract the velocities and flow from US data. For all participants, PWD data were used to extract the velocity over time. To convert this to a flow pulse, the radius of the vessel was extracted using the CD (volunteers) or PWD (patients) data. Furthermore, for the volunteers, the velocity profile over the cross-section was extracted from the CD data

US Doppler images were acquired by experienced sonographers using a Philips Epiq system equipped with a C5-1 probe. Pulsed-Wave Doppler (PWD) and Color Doppler (CD) data were recorded in the longitudinal and transverse plane (Fig. 5), respectively, as proximal as possible while not suffering from acoustic shadowing by the ribs. All acquisitions were obtained in supine position during shallow inspiration breath hold for 3–8 cardiac cycles and the velocity scales were tuned to prevent aliasing. The PWD data were acquired using angle correction with an angle of $\hbox {60}^{\circ }$ and a sample area equal to the vessel diameter, resulting in frame rates of 20–79 Hz and a spatial resolution of 0.25$-$0.46 mm. The CD gain was optimized to obtain as much CD information as possible during diastole, without over-amplifying the signal during systole. Color Doppler frame rates and spatial resolution varied between 7–23 Hz and 0.16$-$0.24 mm, respectively. Additionally, for the patients, 4D US recordings of the aneurysm region were obtained using an X6-1 matrix probe as described by Maas et al. [21]. Finally, directly after the US acquisitions, the brachial blood pressure was measured using an arm cuff.

#### Magnetic resonance imaging

**Fig. 6:**
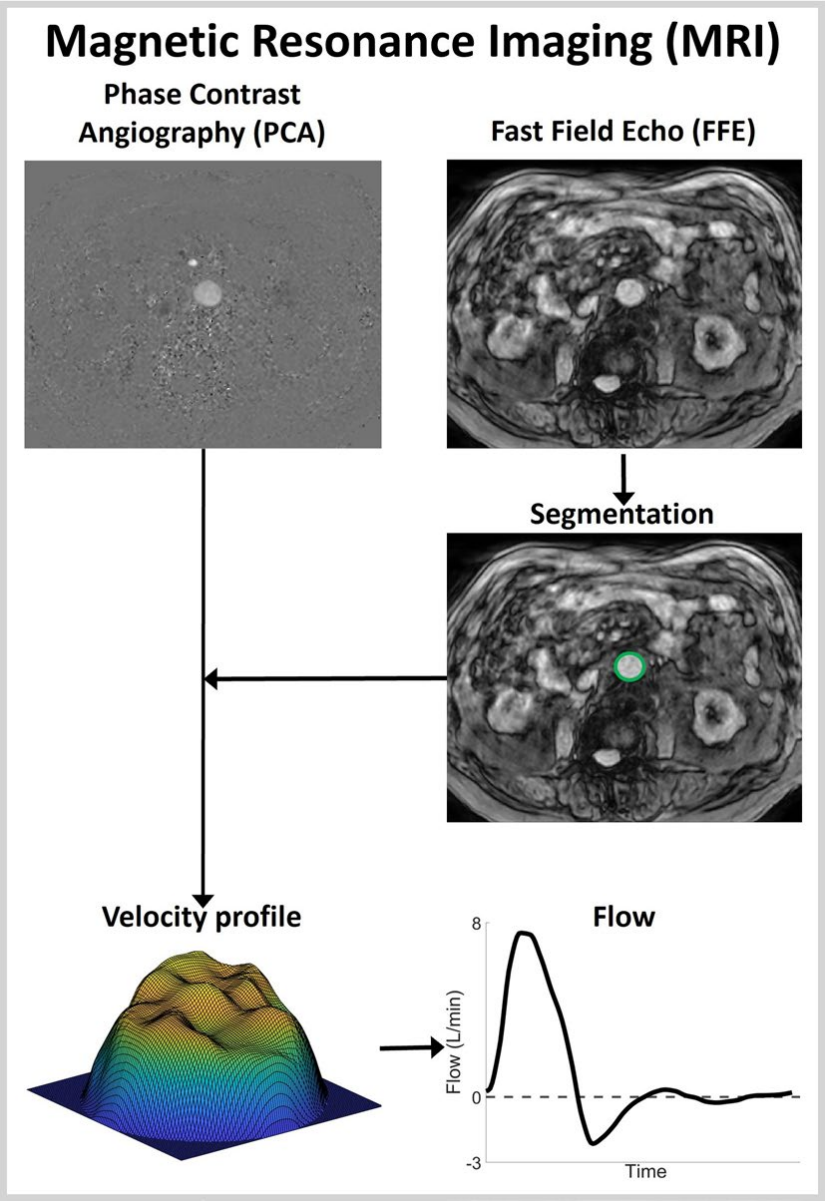
Illustration of the method to extract the velocities from MRI data. The FFE data were used to segment the vessel wall. This segmentation was subsequently used on the PCA data to extract the velocity profile over the cross-section. Integrating the velocity profiles results in the flow pulse

Two-dimensional time-resolved (2D+t) Q-flow MRI images were acquired by experienced MRI technologists, using a fast field echo (FFE) and a phase contract angiography (PCA) sequence (Fig. 6). Clinical 1.5T (volunteers, Philips Ingenia) or 3T (patients, Philips Achieva dStream) scanners were combined with posterior and anterior 16-channel phased array torso coils (Philips dStream), resulting in a spatial resolution of 1.04 mm (volunteers) and 0.96 mm (patients). For the volunteers, the acquisition plane was placed directly distal to the renal arteries, whereas the plane was placed proximal to the aneurysm region in patients, in accordance with the ultrasound acquisition. In all cases, the acquisition plane was positioned perpendicular to the aorta. The velocity encoding (VENC) was based on the maximum velocity as measured with PWD, adjusted if aliasing was observed and ranged between 60–150 cm/s. Data were acquired during shallow inspiration breath hold for 27–64 s and averaged using retrospective ECG gating, resulting in 30 frames over the cardiac cycle. Directly after the MRI scan, the brachial blood pressure was measured using an arm cuff. For one patient (AAA6), the 2D+t MRI data suffered from severe motion artifacts. For this patient, additional 4D Q-flow MRI data were available, with a less optimal spatial resolution of 1.46 mm and temporal resolution of 16 frames over the cardiac cycle. A slice proximal to the aneurysm region was extracted for further analysis, which revealed no motion artifacts.

### Velocity and flow extraction

After acquisition, the US and MRI data were anonymized and further processed in MATLAB (R2023b) to obtain the velocity profile over the cross-section and the flow pulse.

#### Ultrasound

The PWD data were used to obtain the flow pulse (Fig. 5). The PWD spectrum was smoothed with a 3x3 pixel mean filter, after which the velocity for each line in the spectrum was calculated as the weighted median velocity using the pixel intensities as weights. The velocity was determined by calculating the median instead of the mean, since median values are more robust to outliers. Subsequently, the velocity signal was low-pass filtered, split in separate cardiac cycles, averaged, and interpolated using the shape-preserving piece-wise cubic (pchip) method. To preserve the dynamical properties of the velocity profiles of the separate cycles, the profiles were averaged over the shortest heartbeat period and interpolated for the average period time [45]. The resulting velocity profile (*v*) was converted to a flow pulse (*Q*) using the average radius of the aorta (*r*), assuming a symmetric velocity profile and a circular cross-section: $Q = v \cdot \pi r^{2}$.

For the volunteers, the average radius was obtained from the CD data. To this end, the color was removed from the CD data and the vessel was manually selected in the first frame. Subsequently, an in-house developed star-Kalman algorithm was used to segment the vessel over all frames [46, 47]. The resulting segmentation was smoothed by taking a Gaussian-weighted average over all frames, and subsequently used to calculate the average radius. For each frame, the relative velocities were extracted from the CD data by matching the color of each pixel within the segmentation to the color scale. When the segmentation was not completely filled with color information, which often occurs in diastole, the missing data were interpolated. Based on the mean velocity, individual cardiac cycles were extracted and the average velocity profile for each frame was calculated. Lastly, the relative velocities were converted to absolute velocities based on the PWD flow.

However, since the analysis for volunteers showed that it is not feasible to employ CD data to obtain the velocity profile over the cross-section (sect. Velocity profiles over cross-section), the average radius was extracted from the PWD data for the patients. To this end, the upper and lower wall were manually selected on 5 lines perpendicular to the vessel centerline. The radius was then calculated as the average of the Euclidean distance between upper and lower points.

#### Magnetic resonance imaging

The FFE sequence contains anatomical information and is therefore used to segment the vessel wall, using the same star-Kalman method as used for CD (sect. Ultrasound), applied to the complement of the FFE image. The PCA data contain pixel intensities (*I*) that can directly be converted to velocities using the VENC. For each frame, the velocities within the aorta were extracted using the FFE-based segmentation. Subsequently, the velocities were interpolated to a finer grid, smoothed with a 3x3 pixel mean filter and the borders were interpolated to ensure a smooth transition to zero velocity at the border. From the velocity profile over the cross-section, the flow at each frame was calculated by integrating the velocities over the vessel area. Finally, the flow pulse was low-pass filtered and interpolated using again the pchip method.

### Velocity and flow comparison

**Fig. 7:**
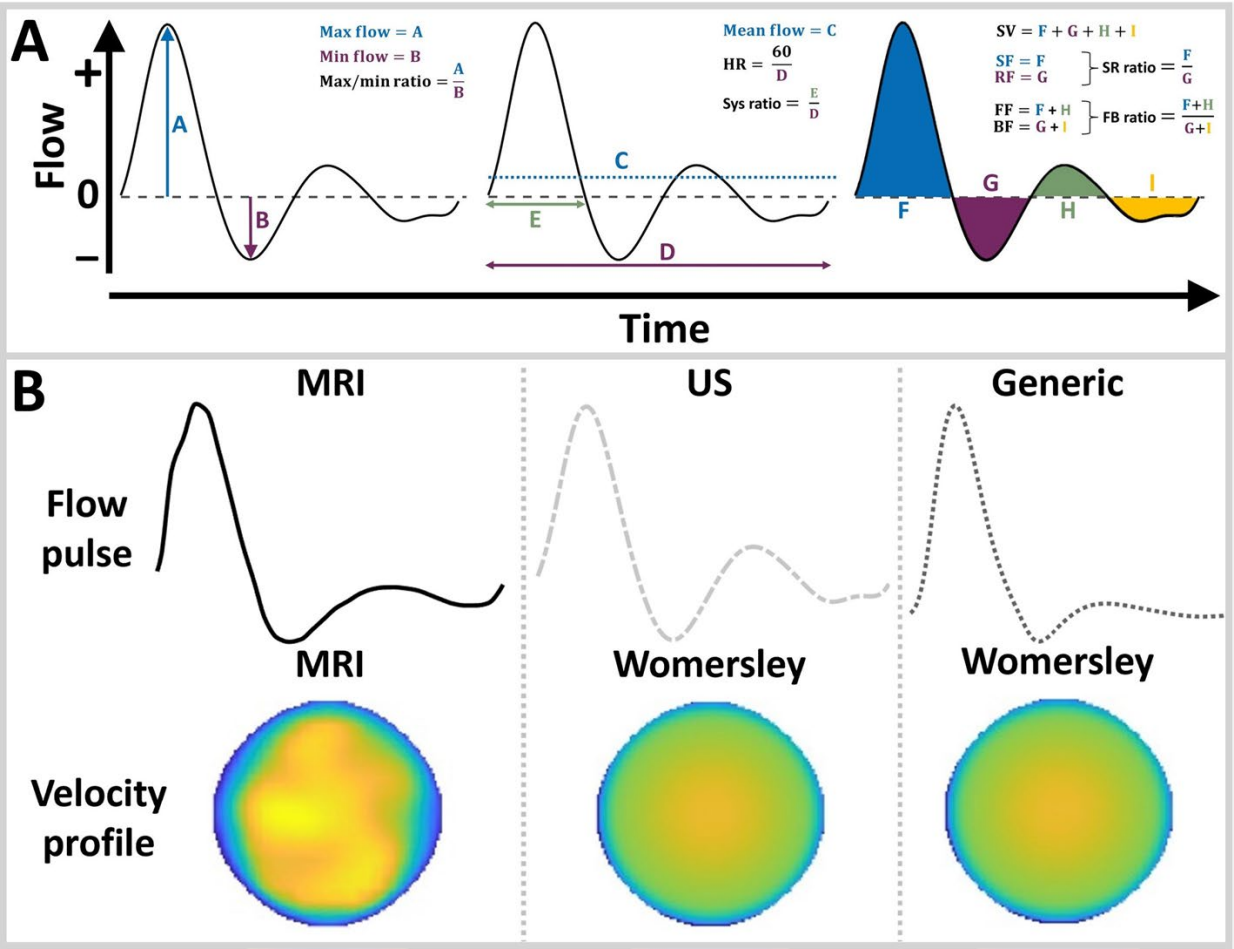
**A** Illustration of the various flow parameters extracted from the flow pulse. **B** Overview of the velocity boundary conditions for the different CFD simulations

To investigate if US velocity profiles and flow pulses would outperform generic alternatives with respect to MRI, generic profiles were also considered for comparison. For the velocity profile over the cross-section, a Womersley profile was employed. The Womersley profile was chosen, based on a comparison between the Womersley, power law, flat (plug), and parabolic profile, as described in Supplementary Section S1. For the flow pulse, the generic profile was obtained using the 1D wave propagation model of Bessems et al. [23, 27, 48], assuming a heart rate of 63.5 beats/min (mean heart rate of all volunteers). The obtained generic profile corresponds well with flow pulses reported in literature [12, 36].

Percentual point-wise differences in velocities with respect to MRI were calculated for the US and Womersley velocity profiles over the cross-section at systole, according to formula 1:

1 $$\begin{aligned} \delta p_{\phi } = \frac{\phi _{US/gen} - \phi _{MRI}}{{\overline{\phi }}_{MRI}} \cdot 100\%, \end{aligned}$$

with $\phi _{US/gen}$ the quantity resulting from the US or generic case and $\phi _{MRI}$ the quantity resulting from the MRI case. In this case, the velocity is the quantity of interest.

For the flow pulses, 13 parameters were defined to quantify the flow (Fig. 7A). Percentual differences in flow parameters with respect to MRI were calculated for the US and generic flow pulses.

### Hemodynamics

To examine the combined effect of the velocity profile and flow pulse on the computed hemodynamics, computational fluid dynamics (CFD) simulations were performed in Ansys Fluent (Ansys Inc., Canonsburg, PA, USA, 2023R1). CFD was preferred over FSI, due to the reduced computational costs. In this study, only the differences in hemodynamics, so not the absolute values, were investigated. As shown in previous studies, CFD simulations result in an overestimation of the WSS compared to FSI simulations, but yield similar spatial patterns, and can therefore be employed to evaluate differences in hemodynamics between different cases [14, 27]. For the volunteers, the aorta was modeled as a straight tube with the MRI-derived radius and a length of 15 cm, complemented by a parametric bifurcation [27]. For the patients, the 4D US data were utilized to obtain the patient-specific AAA geometry using the automatic segmentation method as described by Maas et al. [21]. In proximal direction, the segmentations were elongated by 3 cm. In distal direction, a parametric bifurcation geometry was added [27]. The aortic geometries were converted into triangular surface meshes with a mesh size of 0.4 mm. The volume mesh consisted of 4 layers of prism elements with aspect ratios of 1 with an increase of 20% in element size for each layer. The interior was meshed using tetrahedral elements with an increase in element size of 20% and a maximum element size of 1.4 mm. A mesh sensitivity analysis was performed as part of a previous study [27]. A no-slip condition was assigned to the lumen wall and the shear-thinning behavior of blood was modeled using the Carreau model [49]. At the outlet(s), a 3-element Windkessel model, consisting of a characteristic impedance, peripheral resistance, and an arterial compliance, was used to prescribe the pressure. For each outlet, the Windkessel parameters were determined using geometrical and mechanical properties of the outlet, and the mean pressure and outlet flow. The parameters were iteratively optimized to match the patient-specific blood pressure. A more detailed description of the CFD model can be found in Fonken et al. [27].

For each participant, three CFD simulations were performed in which the inlet flow conditions differed as illustrated in Fig. 7B. For the MRI case, both the flow pulse as the velocity profile over the cross-section were patient-specific. For the US case, only the flow pulse was made patient-specific, since the analysis for volunteers showed that it is not feasible to employ CD data to obtain the velocity profile over the cross-section (section Velocity profiles over cross-section). Lastly, the generic case represents the situation in which no patient-specific velocity information was included in the simulation. In this case, the Womersley profile over the cross-section and the generic flow pulse, as described in section Velocity and flow comparison, were used. Additionally, for the volunteers, the separate impact of the velocity profile over the cross-section on the hemodynamics was investigated by performing simulations with the MRI and Womersley velocity profile, with the same (MRI) flow pulse.

In all cases, two cardiac cycles were simulated and the systolic wall shear stress (systolic WSS), time-averaged wall shear stress (TAWSS, Eq. 2), and oscillatory shear index (OSI, Eq. 3) were evaluated on the last cycle [23]:

2 $$\begin{aligned} TAWSS= & {} \frac{1}{T} \int _{t-T}^{t} |WSS| \ dt, \end{aligned}$$

3 $$\begin{aligned} OSI= & {} \frac{1}{2}(1 - \frac{|\frac{1}{T}\int _{t-T}^{t} WSS \ dt|}{\frac{1}{T} \int _{t-T}^{t} |WSS| \ dt} ). \end{aligned}$$

For the US and generic cases, the percentual point-wise differences with respect to MRI were calculated for each quantity according to Eq. 1 [27]. Additionally, the normalized root-mean-square error (nRMSE) with respect to MRI was calculated using Eq. 4:

4 $$\begin{aligned} nRMSE = \frac{\sqrt{\frac{1}{N} \sum _{i=1}^{N}(\phi _{MRI}^{i} - \phi _{US/gen}^{i})^2}}{{\overline{\phi }}_{MRI}} \cdot 100\%. \end{aligned}$$

For the nRMSE calculation, the most proximal part of the domain, equal to one diameter, was excluded.

### Supplementary Information

Supplementary file 1.

## Data Availability

The datasets used and/or analyzed during the current study are available from the corresponding author on reasonable request.

## References

[1] Salman HE, Ramazanli B, Yavuz MM, Yalcin HC. Biomechanical investigation of disturbed hemodynamics-induced tissue degeneration in abdominal aortic aneurysms using computational and experimental techniques. Front Bioeng Biotechnol. 2019;7:111. 31214581 10.3389/fbioe.2019.00111 PMC6555197

[2] Kontopodis N, Tzirakis K, Tavlas E, Lioudaki S, Ioannou C. Biomechanic and hemodynamic perspectives in abdominal aortic aneurysm rupture risk assessment. London: IntechOpen; 2018.

[3] Lederle FA, Wilson SE, Johnson GR, Reinke DB, Littooy FN, Acher CW, Ballard DJ, Messina LM, Gordon IL, Chute EP, Krupski WC, Busuttil SJ, Barone GW, Sparks S, Graham LM, Rapp JH, Makaroun MS, Moneta GL, Cambria RA, Makhoul RG, Eton D, Ansel HJ, Freischlag JA, Bandyk D. Immediate repair compared with surveillance of small abdominal aortic aneurysms. New Engl J Med. 2002;346(19):1437–44. PMID: 12000813. 12000813 10.1056/NEJMoa012573

[4] Collin J. Uk small aneurysms trial. Lancet. 1999;353(9150):407–8. 10.1016/S0140-6736(05)74979-5 9950466

[5] van Disseldorp EMJ, Petterson NJ, Rutten MCM, van de Vosse FN, van Sambeek MRHM, Lopata RGP. Patient specific wall stress analysis and mechanical characterization of abdominal aortic aneurysms using 4d ultrasound. Eur J Vasc Endovas Surg. 2016;52(5):635–42. 10.1016/j.ejvs.2016.07.088 27665991

[6] Chaikof EL, Dalman RL, Eskandari MK, Jackson BM, Lee WA, Mansour MA, Mastracci TM, Mell M, Murad MH, Nguyen LL, Oderich GS, Patel MS, Schermerhorn ML, Starnes BW. The society for vascular surgery practice guidelines on the care of patients with an abdominal aortic aneurysm. J Vasc Surg. 2018;67(1):2-77.e2. 29268916 10.1016/j.jvs.2017.10.044

[7] Xenos M, Bluestein D. Biomechanical aspects of abdominal aortic aneurysm (aaa): Fluid structure interaction (fsi) studies of aaa behavior. In: McLoughlin T, editor. Biomechanics and mechanobiology of aneurysms. Studies in mechanobiology, tissue engineering and biomaterials. Freiburg: Verlag; 2011.

[8] Fillinger MF, Marra SP, Raghavan ML, Kennedy FE. Prediction of rupture risk in abdominal aortic aneurysm during observation: Wall stress versus diameter. J Vasc Surg. 2003;37(4):724–32. 12663969 10.1067/mva.2003.213

[9] Vorp D. Biomechanics of abdominal aortic aneurysm. J Biomech. 2007;40(02):1887–902. 17254589 10.1016/j.jbiomech.2006.09.003 PMC2692528

[10] Zambrano BA, Gharahi H, Lim C, Jaberi FA, Choi J, Lee W, Baek S. Association of intraluminal thrombus, hemodynamic forces, and abdominal aortic aneurysm expansion using longitudinal ct images. Ann Biomed Eng. 2016;44(5):1502–14. 26429788 10.1007/s10439-015-1461-x PMC4826625

[11] Biasetti J, Gasser T, Auer M, Hedin U, Labruto F. Hemodynamics of the normal aorta compared to fusiform and saccular abdominal aortic aneurysms with emphasis on a potential thrombus formation mechanism. Ann Biomed Eng. 2010;38(02):380–90. 19936925 10.1007/s10439-009-9843-6

[12] Les A, Shadden S, Figueroa C, Park J, Tedesco M, Herfkens R, Dalman R, Taylor C. Quantification of hemodynamics in abdominal aortic aneurysms during rest and exercise using magnetic resonance imaging and computational fluid dynamics. Ann Biomed Eng. 2010;38(02):1288–313. 20143263 10.1007/s10439-010-9949-x PMC6203348

[13] Boyd AJ, Kuhn DCS, Lozowy RJ, Kulbisky GP. Low wall shear stress predominates at sites of abdominal aortic aneurysm rupture. J Vasc Surg. 2016;63(6):1613–9. 25752691 10.1016/j.jvs.2015.01.040

[14] Lin S, Han X, Bi Y, Ju S, Gu L. Fluid-structure interaction in abdominal aortic aneurysm: effect of modeling techniques. BioMed Res Int. 2017;1–10(01):2017. 10.1155/2017/7023078 PMC5340988 28321413

[15] Scotti C, Jimenez J, Muluk S, Finol E. Wall stress and flow dynamics in abdominal aortic aneurysms: finite element analysis vs. fluid-structure interaction. Comput Methods Biomech Biomed Eng. 2008;11(07):301–22. 10.1080/10255840701827412 18568827

[16] Scotti C, Shkolnik A, Muluk S, Finol E. Fluid-structure interaction in abdominal aortic aneurysms: effects of asymmetry and wall thickness. Biomed Eng Online. 2005;4(02):64. 16271141 10.1186/1475-925X-4-64 PMC1298313

[17] Di Martino E, Guadagni G, Fumero A, Ballerini G, Spirito R, Biglioli P, Redaelli A. Fluid-structure interaction within realistic 3d models of the aneurysmatic aorta as a guidance to assess the risk of rupture of the aneurysm. Med Eng Phys. 2001;23(12):647–55. 11755809 10.1016/S1350-4533(01)00093-5

[18] Wolters BJBM, Rutten MCM, Schurink GWH, Kose U, de Hart J, van de Vosse FN. A patient-specific computational model of fluid-structure interaction in abdominal aortic aneurysms. Med Eng Phys. 2005;27(10):871–83. 16157501 10.1016/j.medengphy.2005.06.008

[19] Scotti CM, Finol EA. Compliant biomechanics of abdominal aortic aneurysms: a fluid-structure interaction study. Comput Struct. 2007;85(11):1097–113. 10.1016/j.compstruc.2006.08.041

[20] van Disseldorp EMJ, Petterson NJ, van de Vosse FN, van Sambeek MRHM, Lopata RGP. Quantification of aortic stiffness and wall stress in healthy volunteers and abdominal aortic aneurysm patients using time-resolved 3D ultrasound: a comparison study. Eur Heart J Cardiovasc Imag. 2018;20(2):185–91. 10.1093/ehjci/jey051 29618036

[21] Maas EJ, Nievergeld AHM, Fonken JHC, Thirugnanasambandam M, van Sambeek MRHM, Lopata RGP. 3d-ultrasound based mechanical and geometrical analysis of abdominal aortic aneurysms and relationship to growth. Ann Biomed Eng. 2023;51(07):2554–65. 37410199 10.1007/s10439-023-03301-2 PMC10598132

[22] Nievergeld AHM, Maas EJ, de Ruijter J, Fonken JHC, van Sambeek MRHM, Lopata RGP. Automatic segmentation and mechanical characterisation of the intraluminal thrombus and arterial wall of abdominal aortic aneurysms using time resolved 3d ultrasound images. Eur J Vasc Endovasc Surg. 2022;66(03):418–27. 10.1016/j.ejvs.2023.03.033 36963747

[23] Fonken JHC, Maas EJ, Nievergeld AHM, van Sambeek MRHM, van de Vosse FN, Lopata RGP. Ultrasound-based fluid-structure interaction modeling of abdominal aortic aneurysms incorporating pre-stress. Front Physiol. 2021;12:717593. 34483971 10.3389/fphys.2021.717593 PMC8414835

[24] van Disseldorp EMJ, van Dronkelaar JJ, Pluim JPW, van de Vosse FN, van Sambeek MRHM, Lopata RGP. Ultrasound based wall stress analysis of abdominal aortic aneurysms using multiperspective imaging. Eur J Vasc Endovasc Surg. 2019;59:81–91. 31727437 10.1016/j.ejvs.2019.01.026

[25] Sjoerdsma M, Verstraeten SCFPM, Maas EJ, van de Vosse FN, van Sambeek MRHM, Lopata RGP. Spatiotemporal registration of 3-d multi-perspective ultrasound images of abdominal aortic aneurysms. Ultrasound Med Biol. 2023;49(01):318–32. 36441033 10.1016/j.ultrasmedbio.2022.09.005

[26] van Disseldorp EMJ, Hobelman KH, Petterson NJ, van de Vosse FN, van Sambeek MRHM, Lopata RGP. Influence of limited field-of-view on wall stress analysis in abdominal aortic aneurysms. J Biomech. 2016;49(12):2405–12. 26924662 10.1016/j.jbiomech.2016.01.020

[27] Fonken JHC, Maas EJ, Nievergeld AHM, van Sambeek MRHM, van de Vosse FN, Lopata RGP. The impact of a limited field-of-view on computed hemodynamics in abdominal aortic aneurysms: evaluating the feasibility of completing ultrasound segmentations with parametric geometries. Ann Biomed Eng. 2023;51:1296–309. 36709232 10.1007/s10439-022-03133-6 PMC10172266

[28] Teabi A, Sandler RH, Kakavand B, Mansy HA. Extraction of peak velocity profiles from doppler echocardiography using image processing. Bioengineering. 2019;6:09. 10.3390/bioengineering6030064 PMC6784240 31357566

[29] Fraser KH, Meagher S, Blake JR, Easson WJ, Hoskins PR. Characterization of an abdominal aortic velocity waveform in patients with abdominal aortic aneurysm. Ultrasound Med Biol. 2008;34:73–80. 17689855 10.1016/j.ultrasmedbio.2007.06.015

[30] Chandra S, Raut SS, Jana A, Biederman RW, Doyle M, Muluk SC, Finol EA. Fluid-structure interaction modeling of abdominal aortic aneurysms: the impact of patient-specific inflow conditions and fluid/solid coupling. J Biomech Eng. 2013;135:08. 10.1115/1.4024275 PMC3705803 23719760

[31] Madhavan S, Kemmerling EMC. The effect of inlet and outlet boundary conditions in image-based cfd modeling of aortic flow. BioMed Eng Online. 2018;17:17–66. 29843730 10.1186/s12938-018-0497-1 PMC5975715

[32] Tzirakis K, Kamarianakis Y, Kontopodis N, Ioannou CV. The effect of blood rheology and inlet boundary conditions on realistic abdominal aortic aneurysms under pulsatile flow conditions. Bioengineering. 2023;10:02. 10.3390/bioengineering10020272 PMC9953019 36829766

[33] Kose U, de Putter S, Hoogeveen R, Breeuwer M. Computational fluid dynamics of abdominal aortic aneurysms with patient-specific inflow boundary conditions. Proc SPIE, Med Imag. 2006;135:03.

[34] Bollache E, van Ooij P, Powell A, Carr J, Markl M, Barker AJ. Comparison of 4d flow and 2d velocity-encoded phase contrast mri sequences for the evaluation of aortic hemodynamics. Int J Cardiovasc Imag. 2016;32:10. 10.1007/s10554-016-0938-5 PMC5096729 27435230

[35] Wentland AL, Grist TM, Wieben O. Repeatability and internal consistency of abdominal 2d and 4d phase contrast mr flow measurements. Acad Radiol. 2013;20:06. 10.1016/j.acra.2012.12.019 PMC3897393 23510798

[36] Cheng C, Herfkens R, Taylor C. Comparison of abdominal aortic hemodynamics between men and women at rest and during lower limb exercise. J vasc surg. 2003;37(01):118–23. 12514587 10.1067/mva.2002.107

[37] Mitchell DG. Color doppler imaging: principles, limitations, and artifacts. Radiology. 1990;177(1):1–10. 10.1148/radiology.177.1.2204956 2204956

[38] von Bibra H, Stempfle HU, Poll A, Scherer M, Blüml G, Blömer H. Limitations of flow detection by color doppler: in vitro comparison to conventional doppler. Echocardiography. 1991;8(6):633–42. 10149274 10.1111/j.1540-8175.1991.tb01025.x

[39] Baun Jim. Emerging technology: ultrasound vector flow imaging-a novel approach to arterial hemodynamic quantification. J Diagn Med Sonogr. 2021;37(6):599–606. 10.1177/87564793211036013

[40] Antonuccio MN, Morales HG, This A, Capellini K, Avril S, Celi S, Rouet L. Towards the 2d velocity reconstruction in abdominal aorta from color-doppler ultrasound. Med Eng Phys. 2022;107: 103873. 36068045 10.1016/j.medengphy.2022.103873

[41] Pelc Norbert J. Flow quantification and analysis methods. Magn Reson Imag Clin North Am. 1995;3(3):413–24. 10.1016/S1064-9689(21)00253-1 7584247

[42] Nayak KS, Nielsen JF, Bernstein MA, Markl M, Gatehouse PD, Botnar RM, Saloner D, Lorenz C, Wen H, Hu BS, Epstein FH, Oshinski JN, Raman SV. Cardiovascular magnetic resonance phase contrast imaging. J Cardiovasc Magn Reson. 2015;17:71. 26254979 10.1186/s12968-015-0172-7 PMC4529988

[43] Lisanti Christopher J, Douglas David B. Effects of breath-hold and cardiac cycle on the mri appearance of the aorta and inferior vena cava in t2 abdominal imaging. Am J Roentgenol. 2009;192(5):1348–58. PMID: 19380560. 19380560 10.2214/AJR.08.1646

[44] Sakuma Hajime, Kawada Nanaka, Kubo Hitoshi, Nishide Yoshiya, Takano Katsuhiro, Kato Noriyuki, Takeda Kan. Effect of breath holding on blood flow measurement using fast velocity encoded cine mri. Magn Reson Med. 2001;45(2):346–8. 11180443 10.1002/1522-2594(200102)45:2<346::AID-MRM1044>3.0.CO;2-I

[45] Leguy C. *On the clinical estimation of the hemodynamical and mechanical properties of the arterial tree*. PhD thesis, Eindhoven University of Technology, 2010.

[46] Guerrero J, Salcudean S, Mcewen J, Masri B, Nicolaou S. Real-time vessel segmentation and tracking for ultrasound imaging applications. IEEE Trans Med Imag. 2007;26(09):1079–90. 10.1109/TMI.2007.899180 17695128

[47] de Ruijter J, van Sambeek M, van de Vosse F, Lopata R. Automated 3d geometry segmentation of the healthy and diseased carotid artery in free-hand, probe tracked ultrasound images. Med Phys. 2020;47(3):1034–47. 31837022 10.1002/mp.13960 PMC7079173

[48] Bessems D, Rutten M, Van De Vosse F. A wave propagation model of blood flow in large vessels using an approximate velocity profile function. J Fluid Mech. 2007;580:145–68. 10.1017/S0022112007005344

[49] Adélia Sequeira, João Janela. An overview of some mathematical models of blood rheology. Berlin: Springer; 2007. p. 65–87.

