# Supplementary material for: On the feasibility of ultrasound Doppler-based personalized hemodynamic modeling of the abdominal aorta
Source: Biomed Eng Online. 2024 Jul 25;23:71. doi: 10.1186/s12938-024-01267-3 (PMC11270776; doi:10.1186/s12938-024-01267-3)
Supplement: Supplementary file 1 [file 12938_2024_1267_MOESM1_ESM.pdf]

Supplementary material

S1 Choice of generic velocity profile

In our previous research [14, 15], a parabolic velocity profile was assumed. However, various other papers have shown the impact of the velocity profile on the hemodynamics [8,25,37], but their results (partially) contradict. Therefore, for each volunteer, 4 CFD simulations were performed with the patient-specific MRI-based flow pulse, but a different generic velocity profile over the cross-section: Womersley, powerlaw, flat (plug) or parabolic. The powerlaw profile largely resembles the flat profile, except for the boundaries of the domain. For the flat profile, the velocities at the boundaries equal the velocities in the rest of the domain, whereas for the powerlaw profile, the velocities at the borders are set to zero, with a steep increase in velocity near the boundaries. The nRMSE for the systolic WSS, TAWSS and OSI are summarized in Figure S1. In the left column, the complete vessel was taken into account while calculating the nRMSE, whereas in the right column, the first 4 diameters were excluded from the calculation.

For the systolic WSS and TAWSS, the parabolic profile results in the smallest nRMSE in the complete vessel, followed by the Womersley profile. However, when excluding the inlet region, the parabolic profile yields the largest error, whereas the Womersley profile results in the smallest error. For the OSI, the Womersley profile performs best, both in the complete domain as in the part excluding the inlet. In the latter, the differences between the different profiles are most pronounced, with the parabolic profile performing the worst.

This analysis demonstrates that the Womersley profile is the most favorable option. Therefore, it is chosen as the generic profile for all other simulations.

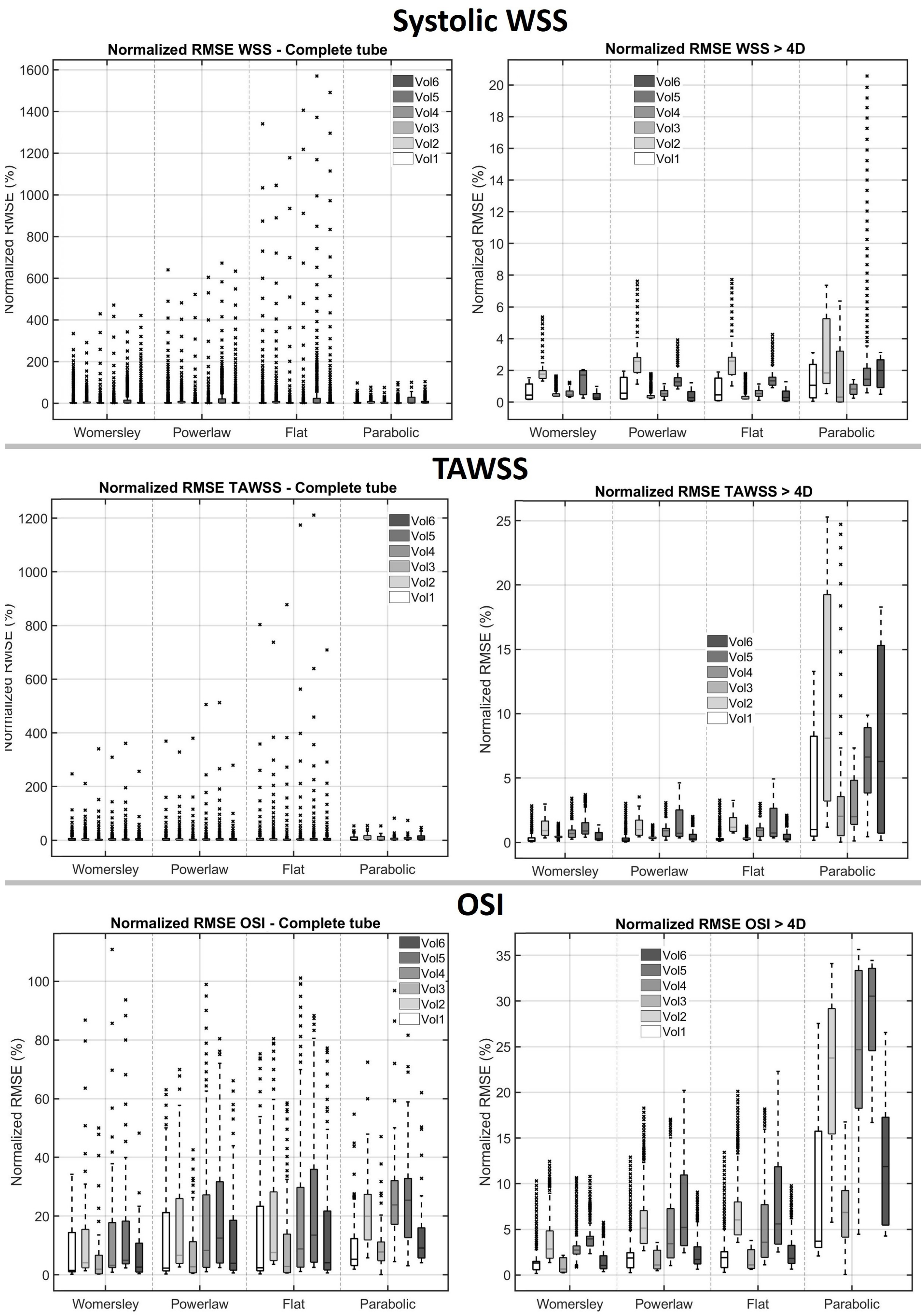

Figure S1: The nRMSE for the 4 different generic velocity profiles for the complete vessel (left column) and the vessel, excluding the first 4 diameters (right column) for the systolic WSS (top), TAWSS (middle) and OSI (bottom).

S2 Additional figures

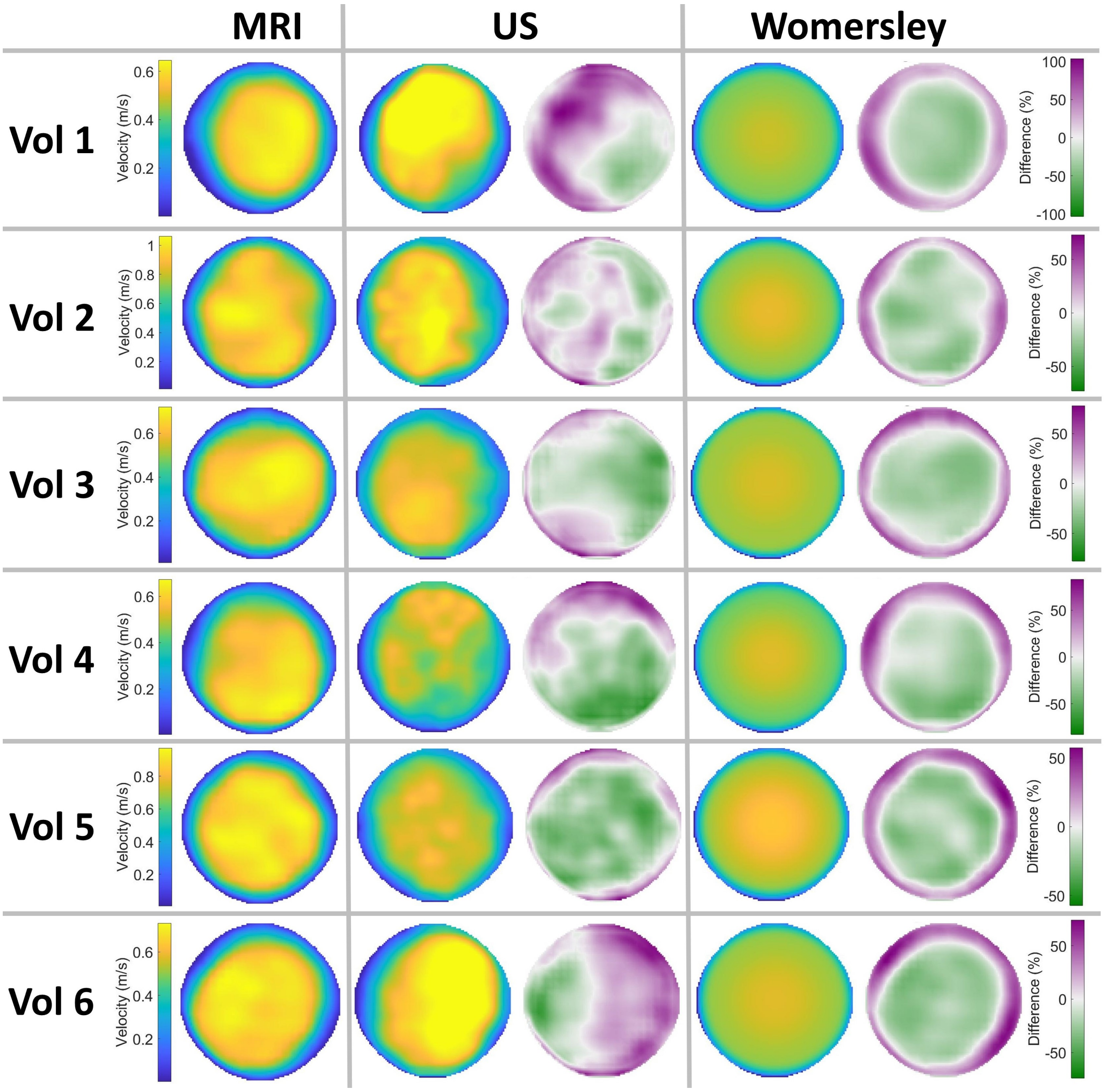

Figure S2: Systolic MRI, US and Womersley velocity profiles over the cross-section (blue-yellow), and their percentual differences (green-purple), for all volunteers.

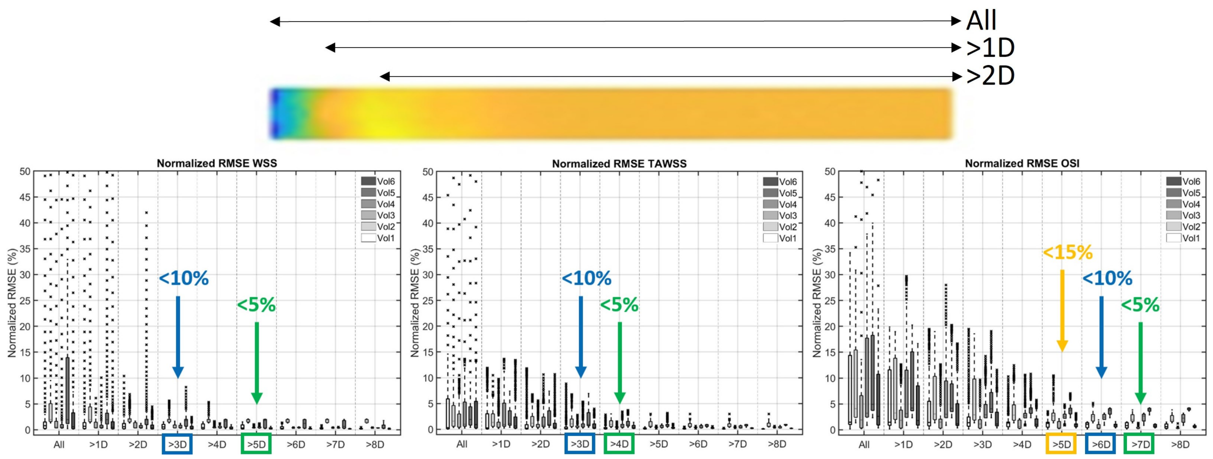

Figure S3: NRMSE of systolic WSS, TAWSS and OSI over the length of the vessel. On top, the nomenclature is schematically explained. "All" means that the complete tube was considered when calculating the nRMSE. ">1D" means that the first diameter was excluded from the calculation, ">2D" means that the first 2 diameters were excluded, etc.
